# Early childhood linear growth faltering in low-income and middle-income countries as a whole-population condition: analysis of 179 Demographic and Health Surveys from 64 countries (1993–2015)

**DOI:** 10.1016/S2214-109X(17)30418-7

**Published:** 2017-11-10

**Authors:** Daniel E Roth, Aditi Krishna, Michael Leung, Joy Shi, Diego G Bassani, Aluisio J D Barros

**Affiliations:** a Department of Pediatrics, Hospital for Sick Children and University of Toronto, Toronto, ON, Canada; b Centre for Global Child Health and SickKids Research Institute, Hospital for Sick Children, Toronto, ON, Canada; c Harvard T H Chan School of Public Health, Boston, MA, USA; d International Center for Equity in Health, Universidade Federal de Pelotas, Pelotas, Brazil

## Abstract

**Background:**

The causes of early childhood linear growth faltering (known as stunting) in low-income and middle-income countries remain inadequately understood. We aimed to determine if the progressive postnatal decline in mean height-for-age *Z* score (HAZ) in low-income and middle-income countries is driven by relatively slow growth of certain high-risk children versus faltering of the entire population.

**Methods:**

Distributions of HAZ (based on WHO growth standards) were analysed in 3-month age intervals from 0 to 36 months of age in 179 Demographic and Health Surveys from 64 low-income and middle-income countries (1993–2015). Mean, standard deviation (SD), fifth percentiles, and 95th percentiles of the HAZ distribution were estimated for each age interval in each survey. Associations between mean HAZ and SD, fifth percentile, and 95th percentile were estimated using multilevel linear models. Stratified analyses were performed in consideration of potential modifiers (world region, national income, sample size, year, or mean HAZ in the 0–3 month age band). We also used Monte Carlo simulations to model the effects of subgroup versus whole-population faltering on the HAZ distribution.

**Findings:**

Declines in mean HAZ from birth to 3 years of age were accompanied by declines in both the fifth and 95th percentiles, leading to nearly symmetrical narrowing of the HAZ distributions. Thus, children with relatively low HAZ were not more likely to have faltered than taller same-age peers. Inferences were unchanged in surveys regardless of world region, national income, sample size, year, or mean HAZ in the 0–3 month age band. Simulations showed that the narrowing of the HAZ distribution as mean HAZ declined could not be explained by faltering limited to a growth-restricted subgroup of children.

**Interpretation:**

In low-income and middle-income countries, declines in mean HAZ with age are due to a downward shift in the entire HAZ distribution, revealing that children across the HAZ spectrum experience slower growth compared to the international standard. Efforts to mitigate postnatal linear growth faltering in low-income and middle-income countries should prioritise action on community-level determinants of childhood HAZ trajectories.

**Funding:**

Bill & Melinda Gates Foundation.

## Introduction

Linear growth faltering—an abnormally slow rate of gain in a child's height or length—is an important aspect of the poor health and social conditions of many children in low-income and middle-income countries.^1^ International comparisons of growth patterns typically use height-for-age *Z* scores (HAZ), derived by age and sex standardisation of each individual child's height mapped onto the standard distributions produced by WHO.^2^ The degree of faltering of a population is expressed as the mean HAZ (relative to a mean of zero in a healthy population) or the prevalence of stunting (ie, proportion of children with HAZ <–2).^1^, ^3^

A consistent observation in low-income and middle-income countries has been a mean HAZ less than 0 at birth followed by a progressive decline in mean HAZ starting in early infancy and continuing throughout the first two years of life.^1^, ^3^ The causes of the observed low mean HAZ and high prevalence of stunting in low-income and middle-income countries have been the subject of many epidemiological studies, primarily focusing on individual or household-level risk factors (ie, exposures that vary between individuals or households within the study population).^1^, ^4^ Many risk factors for stunting have been identified (eg, low maternal education, infections, micronutrient deficiencies),^1^, ^4^ but causal pathways have not been clearly defined. Biological factors with the largest attributable fractions for stunting (eg, fetal growth restriction, maternal height) are not easily modified,^4^ whereas those that are modifiable (eg, micronutrient status) have modest effects.^5^ Community-level social and economic determinants of child nutrition have been increasingly recognised,^1^, ^6^, ^7^ but these are also frequently derived from measures at the household level and included as individual-level covariates in epidemiological studies.^4^ The interpretation of child-level and household-level risk factors for low HAZ (or stunting) as causes of the postnatal decline of mean HAZ with age might be subject to the atomistic fallacy—the error of drawing inferences about causes of variations between groups on the basis of observations of variations between individuals within a group.^8^ Heights differ among individuals within populations due to variations in genetics, environmental exposures, and intergenerational gene–environment interactions (eg, epigenetics),^9^, ^10^ but these same factors might not explain the population HAZ deficit in low-income and middle-income countries relative to international norms.

**Research in context**

**Evidence before this study**

We searched PubMed in May 20, 2017, with no language restrictions, for epidemiological studies of the changes in height-for-age *Z* score (HAZ) distributional parameters associated with postnatal linear growth faltering in low-income and middle-income countries. We used the following search terms: “growth faltering” or “stunting” AND “child*” or “infan*” AND “population” or “demographic and health survey*” or “global” or “developing countries”[MeSH] or “LMIC*”. We also searched for relevant Demographic and Health Survey (DHS) methodological reports. Numerous studies in low-income and middle-income countries have estimated mean HAZ and other parameters of the HAZ distribution (eg, standard deviation [SD]); however, very few studies have focused on variations in the shape or SD of the HAZ distribution. We found one single-site study and two multi-DHS studies that demonstrated higher HAZ SDs in younger versus older children. The latter two multi-DHS studies also examined variations in SD across surveys, primarily as a measure of anthropometric data quality. One of the multi-DHS studies graphically assessed the relationship between SD and mean HAZ across surveys, concluding that there was no association. At least four studies or narrative reviews or commentaries have shown or commented on the apparent whole-population downward shift in HAZ distributions in children younger than 5 years in low-income and middle-income countries. However, we did not find previous empirical studies of the changes in the HAZ distribution that occur in the context of population linear growth faltering in infancy (ie, decline in mean HAZ with age).

**Added value of this study**

Based on nationally representative anthropometric data from 179 Demographic and Health Surveys (DHS) in 64 countries, this study empirically demonstrated that the population-level early childhood decline in mean HAZ in low-income and middle-income countries is associated with a downward shift in the entire HAZ distribution and a decrease in the HAZ SD. The findings suggest that the decline in mean HAZ cannot be attributed to the faltering of only a vulnerable or high-risk subgroup of children.

**Implications of all the available evidence**

The findings of this study and previous studies and commentaries directly challenge an assumption implicit in many epidemiological studies—ie, that the causes of postnatal linear growth faltering in low-income and middle-income countries can be discerned by analysing between-child variations in growth-limiting exposures (stunting risk factors). Low HAZ or stunting (HAZ <–2) does not equate with linear growth faltering in low-income and middle-income countries, since children not classified as stunted have also likely experienced growth deficits. The present findings support the view that the dominant underlying causes of postnatal linear growth faltering are population-wide exposures (ie, community-level or ubiquitous factors to which nearly all children in the population are exposed) rather than distinct behaviours or characteristics of certain individuals or households. Therefore, efforts to address the early burden of childhood undernutrition should prioritise research and action on community-level determinants of child health in low-income and middle-income countries.

In his seminal paper on population health, Geoffrey Rose (1985) wrote that, “To find the determinants of prevalence and incidence rates, we need to study characteristics of populations, not characteristics of individuals.”^11^ Analyses of time trends and between-population differences in the distribution of a continuous health parameter have previously used the dispersion of the parameter, in relation to its mean, to assess whether individuals are equitably or unevenly implicated in changes in the mean.^12^, ^13^, ^14^ For example, Rose and Day^12^ showed, that across 52 populations in 32 countries, means of the distributions of several chronic disease risk factors (eg, blood pressure) were closely correlated with the proportions of people above a conventional threshold (eg, hypertension), suggesting that population-wide factors that shifted the entire distributions were more important than the behaviours of the subgroup of individuals in the upper tails of the distributions. By contrast, cross-sectional surveys of adults in the USA^13^, ^15^ and internationally^14^ have shown that body-mass index (BMI) distributions have widened over time, suggesting that upward trends in mean BMI are at least partly due to inequitable distribution of the causes of obesity among individuals.

We hypothesised that the postnatal decline in mean HAZ in low-income and middle-income countries represents a whole-population downward shift in the HAZ distribution, which would be empirically revealed by a lack of progressive negative skewness or increase in the standard deviation (SD) of the HAZ distribution as the mean HAZ falls. Such a pattern would provide evidence of the exposure of the entire population to community-level social and macroeconomic causes of growth faltering. Conversely, progressive negative dispersion of the HAZ distribution with age would indicate that the decline in mean HAZ was attributable to the faltering of a particular subgroup of growth-restricted children, thereby supporting the prevailing assumption that between-child variation in HAZ also explains the population-average HAZ deficit in low-income and middle-income countries.

To test our hypothesis, we aimed to: describe the pattern of age-related changes in the mean, SD, fifth percentile, and 95th percentile of the HAZ distribution from birth to 3 years in population-based surveys in low-income and middle-income countries; estimate the association between mean HAZ and other properties of the HAZ distribution (ie, SD, fifth percentile, and 95th percentile) in low-income and middle-income countries; and use computer simulation to compare the effects of subgroup versus whole-population faltering on the decline in the population HAZ distribution.

## Methods

Study design and participantsWe conducted a cross-sectional study of selected distributional parameters of HAZ (based on WHO child growth standards^2^) in 179 Demographic and Health Surveys (DHS) from 64 low-income and middle-income countries, from DHS round III (1993–97) to 2015 (appendix pp 3–7). 29 DHS surveys from this period were excluded because they were partial interim surveys, anthropometric data were unavailable, or data access was restricted (appendix pp 8).

DHS surveys vary in design and sampling procedures but adhere to common principles and practices (appendix p 1), as previously described.^16^ Our analysis was restricted to children aged 0–36 months because all surveys produced HAZ scores for this age range, which covers the crucial postnatal period in which most population faltering occurs in low-income and middle-income countries.^3^ For convenience, we use the term height to refer to both height (measured at 24 months or older) or length (<24 months of age) and HAZ for both height-for-age and length-for-age *Z* scores. In the primary analysis, we excluded extreme HAZ values considered to be implausible (>9 and <–9).

DHS surveys are publicly available anonymised datasets; therefore, this study was exempt from institutional ethical review based on national and institutional policies.

### Procedures

Within each survey, children were grouped by 12 discrete 3-month age bands (exact duration of each interval was 91·3 days) based on the child's age at the time of assessment (referred to as survey-age units). We defined the group-level unit of analysis on the basis of 3-month intervals to optimise the number of children within each survey-age unit without aggregating data over an excessively wide age span in which the decline in mean HAZ could inflate the variance in that period. For each survey-age unit, we estimated the following parameters of the HAZ distribution, accounting for DHS probability weights: mean, median, SD, fifth percentile, and 95th percentile. We also derived the distance in absolute HAZ units from the mean to the fifth percentile (Δp5) and the distance between the mean and the 95th percentile (Δp95). The multisurvey dataset consisted of all survey-age units, totalling 2148 datapoints in a three-level, nested, hierarchical structure: survey-age units clustered within surveys and surveys (identified by year) clustered by country. Survey-age units (datapoints) were not weighted to account for different underlying sample sizes (ie, number of children) because we were interested in survey-level associations rather than inferences at the individual child level.

### Statistical analysis

To describe the pattern of age-related changes in the HAZ distribution from birth to 3 years in low-income and middle-income countries, we generated international summary means and 95% CIs for each of the HAZ distributional parameters (mean, median, SD, fifth percentile, and 95th percentile) by pooling all survey-age units within each age band, accounting for clustering by country (appendix p 1). To estimate the association between mean HAZ and other properties of the HAZ distribution, we used multilevel linear models that rely on the natural within-survey variation in mean HAZ with age and account for the non-independence of the survey-age units within each survey and the within-country clustering (appendix p 2). To model the association between mean HAZ and the tails of the distribution, we report estimates using Δp5 and Δp95 as dependent variables to facilitate comparisons of the narrowing or widening of the distribution above and below the mean. To simplify interpretability of estimates in the context of the age-related decline in mean HAZ, we reversed the sign of mean HAZ in all analyses (ie, if SD increased as mean HAZ declined, the coefficient for SD would be positive). We generated stratum-specific estimates by introducing interactions between mean HAZ and each of the following survey-level fixed effects (in separate models): World Bank world region, survey year, World Bank income level (corresponding to the year of the survey), survey size, and mean HAZ in the 0–3 month age group. We conducted sensitivity analyses of the main estimates by restricting to age younger than 24 months (which is the period in which population faltering is predominant^3^), excluding HAZ values that were less than −6 or more than 6 (more stringent plausibility thresholds recommended by WHO), using 1-month age bands, and using 6-month age bands. To explore heterogeneity across surveys, we used fixed effects regression models including interaction terms between survey and mean HAZ to generate forest plots of the survey-specific estimates (and 95% CIs) of the association of SD, fifth percentile, and 95th percentile with mean HAZ.

We used computer simulation to estimate the effects of a dichotomous exposure representing a theoretical set of factors responsible for growth faltering on progressive shifts in the population HAZ distribution. We started with an initial simulated population of 10 000 children with a HAZ distribution matching that of the WHO standard (ie, mean=0, SD=1). Then, the mean HAZ of the population was lowered from 0 to −2 by inducing 20 successive decrements of 0·1 HAZ, under scenarios that differed with respect to the proportion of the population that were exposed (25%, 50%, 75%, or 100%), whereby exposure status of each child was defined at baseline and remained unchanged across successive decrements. Thus, the mean decline at each decrement was the weighted average of the changes in the exposed and unexposed groups; for example, if only 25% of the population were exposed, then this group would have to demonstrate a mean decline of 0·4 at each decrement for the whole population mean to decline by 0·1. We did not assume that the magnitude of decline was uniform among those exposed; rather, individual child declines were allowed to vary randomly at each decrement, but in aggregate yielded the required mean decline with a standard deviation of a tenth of the mean decline at each decrement. Each scenario was run as a Monte Carlo simulation with 1000 replications, across which we averaged the estimated mean, SD, fifth percentile, and 95th percentile of the HAZ distribution at each decrement. In the primary simulation, selection into the exposed groups was random (ie, independent of HAZ). In modifications of the simulation, we preferentially selected children with low HAZ into the exposed group, introduced a floor effect (whereby children who reached HAZ <–6 were considered to have died or been censored), preferentially selected children with high HAZ into the exposed group, and selected children at random into the exposed group but set the magnitude of the HAZ deficit to be higher for children with higher baseline HAZ.

Analyses and simulations were done with Stata version 13.

### Role of the funding source

The funders of the study had no role in study design, data collection, data analysis, data interpretation, or writing of the report. The corresponding author had full access to all the data in the study and had final responsibility for the decision to submit for publication.

## Results

Surveys were broadly representative of all world regions (except North America) from 1993 to 2015 (table 1). Most countries had multiple surveys, but Peru (n=9) and Bangladesh (n=6) were the most frequent contributors. Of 716 281 children with anthropometric data, 7054 (0·99%) extreme HAZ values (>9 or <–9) were excluded from the primary analysis. The number of children in each survey-age unit varied widely, but the median and ranges were consistent across age bands (appendix p 9).

**Table 1:** Characteristics of included Demographic and Health Surveys

		**Characteristics**
Surveys		179
Countries		64
Surveys per country		
	1	16 (25%)
	2	13 (20%)
	>2	35 (55%)
Surveys by calendar period		
	1993–94	8 (4%)
	1995–99	39 (22%)
	2000–04	34 (19%)
	2005–09	45 (25%)
	2010–15	53 (30%)
Surveys by world region		
	East Asia and Pacific	5 (3%)
	Europe and central Asia	15 (8%)
	Latin America and Caribbean	33 (18%)
	Middle East and north Africa	10 (6%)
	South Asia	14 (8%)
	Sub-Saharan Africa	102 (57%)
Surveys by income classification		
	Low income	107 (60%)
	Lower middle income	59 (33%)
	Upper middle income	13 (7%)
Children per survey		
	Median (IQR)	3159 (2495)
	Range	351 to 27352
Mean HAZ in the 0 to <3-month age band		
	Mean (SE*)	−0·44 (0·05)
	Range	−2·07 to 0·79

Region and income classifications according to the World Bank.

* Standard error of the distribution of survey means.

**Figure 1:**
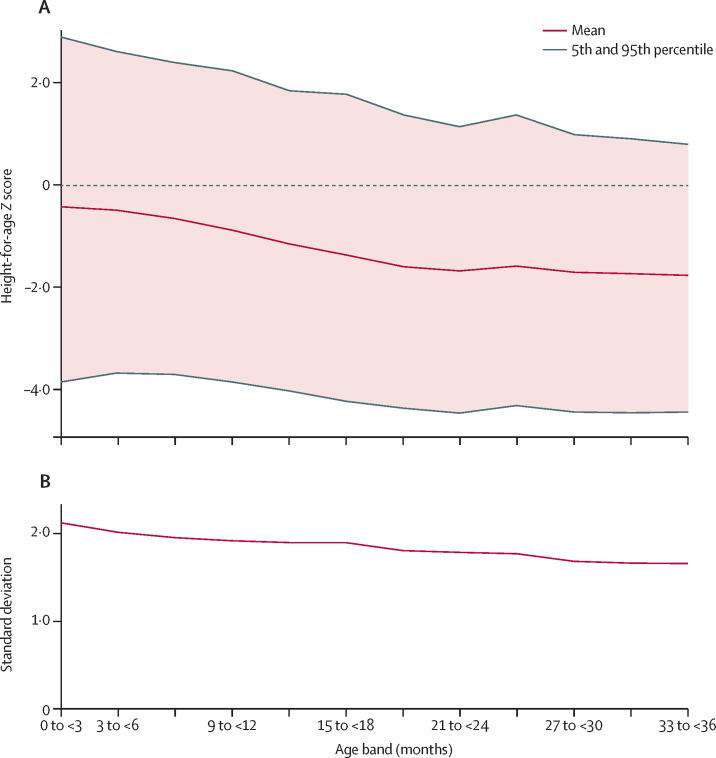
Trends in cross-sectional HAZ distribution parameters for successive 3-month age bands Figure shows data for 2148 survey-age units. (A) Mean, fifth percentile, and 95th percentile. (B) Standard deviation. HAZ=height-for-age *Z* score.

**Figure 2:**
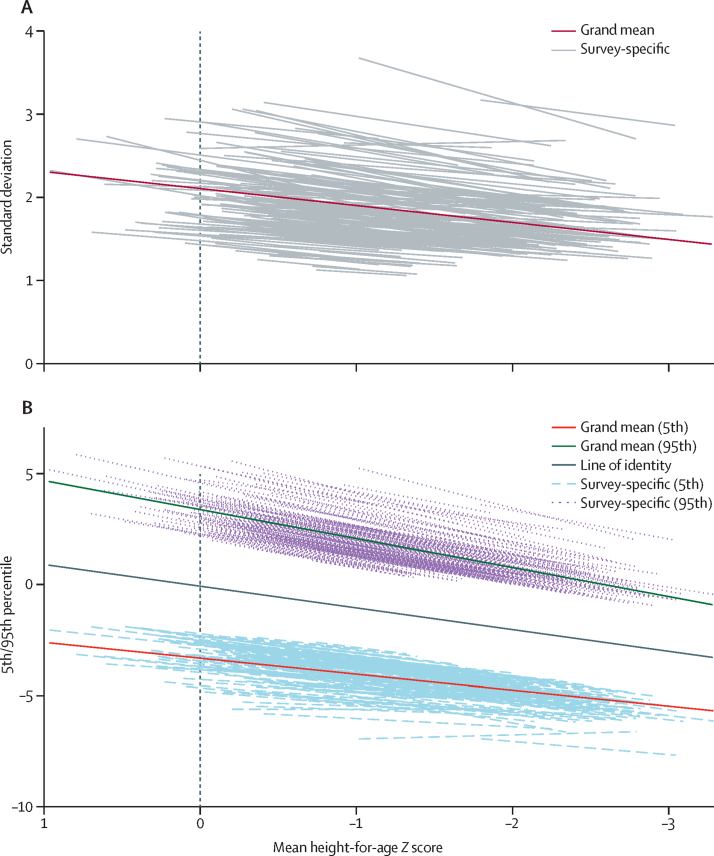
Predicted linear fit lines representing the associations of standard deviation (A) or fifth and 95th percentiles (B) of HAZ distribution with mean HAZ Figure based on multilevel regression models for 2148 survey-age units. Trend lines for individual surveys are best linear unbiased predictions based on survey-level and country-level random intercepts and random slopes at the survey level. In panel A, the two surveys with predicted slopes that most differed from the grand mean slope was Uzbekistan 1996 (n=1086), for which the predicted slope was 0·041, and Guyana 2009 (n=1185), for which the predicted slope was 0·046. No other surveys had predicted slopes for the association of standard deviation with mean HAZ that indicated that standard deviation increased as mean HAZ declined. HAZ=height-for-age *Z* score.

Across all included surveys, from birth to 36 months of age, the age-related decline in population mean HAZ was accompanied by declines in both mean fifth and 95th percentiles (figure 1). The decline in mean fifth percentile was more gradual than the descent of the mean HAZ, whereas mean 95th percentile declined more steeply than mean HAZ (figure 1; appendix p 9). Multilevel regression models showed that, within surveys, the decline in mean HAZ was associated with declines in the SD, median, Δp5, and Δp95 (table 2). The median declined at nearly the same rate as the mean (table 2), consistent with a lack of asymmetric dispersion. The reduction of the SD was due to the contraction of both the upper and lower halves of the distribution, evidenced by the nearly symmetrical convergence of both fifth and 95th percentiles towards the mean HAZ as they declined (table 2, figure 2). Predicted survey-specific slopes were consistent with the direction and magnitude of the overall mean estimates despite substantial variation in the intercepts (figure 2). Separate survey-specific fixed-effects models similarly showed that very few surveys had patterns consistent with an increase in SD as HAZ declined (appendix pp 11–12).

**Table 2:** Associations between distributional parameters and mean HAZ from 0 to 35 months of age

	**Standard deviation**	**Median**	**Δp5**	**Δp95**
Estimated mean change in parameter (95% CI) for a 1-unit decline in mean HAZ (fixed-effect slope)	−0·20 (−0·22 to −0·18)	−0·98 (−0·99 to −0·97)	−0·28 (−0·34 to −0·23)	−0·31 (−0·36 to −0·26)
Estimated mean parameter (95% CI) when mean HAZ=0 (fixed-effect intercept)	2·10 (2·00 to 2·20)	−0·046 (−0·066 to −0·026)	3·29 (3·12 to 3·46)	3·40 (3·19 to 3·61)
Predicted mean parameter (95% CI) when mean HAZ=–2	1·70 (1·61 to 1·79)	−2·01 (−2·03 to −2·00)	2·72 (2·58 to 2·87)	2·79 (2·60 to 2·97)
Intraclass correlation coefficient (95% CI) at the country level*	0·39 (0·27 to 0·53)	0·09 (0·05 to 0·16)	0·27 (0·18 to 0·40)	0·35 (0·23 to 0·48)
Intraclass correlation coefficient (95% CI) at the survey year level to nested within country†	0·86 (0·82 to 0·89)	0·28 (0·20 to 0·38)	0·79 (0·74 to 0·83)	0·78 (0·72 to 0·82)

Table shows data for 2148 survey-age units, based on multilevel linear regression models. HAZ=height-for-age *Z* score. Δp5=distance from the mean to the fifth percentile. Δp95=distance from the mean to the 95th percentile.

* Proportion of variance in the parameter (across survey-age units) that is accounted for by clustering of survey-age units within country. A higher value reflects greater similarity among survey-age units within the same country.

† Proportion of variance in the parameter (across survey-age units) that is accounted for by clustering of survey-age units within surveys (ie, clustering by both country and year of survey). A higher value reflects greater similarity among survey-age units within the same survey.

Findings were generally consistent across subgroups (appendix pp 13–17). Specifically, SD decreased with the decline in mean HAZ in all world regions, all survey years, and all income groups, and irrespective of survey size or mean HAZ at baseline (0–3 months). There were no subgroups in which Δp5 or Δp95 increased as mean HAZ declined. Differences between the paired estimates for Δp5 and Δp95 were generally similar across subgroups; however, the magnitude of the estimates for Δp95 were relatively high compared with Δp5 in Europe and Central Asia region and Middle East and North Africa region and relatively low in east Asia and the Pacific region. There was no strong evidence that a floor effect buffered the decline of the lower tail of the HAZ distribution; specifically, surveys in the lowest tertile of mean HAZ at baseline (0–3 months) did not have higher-magnitude estimates for the Δp5 slope (appendix p 17). Inferences based on main effect estimates were unchanged in all sensitivity analyses (appendix p 10).

**Figure 3:**
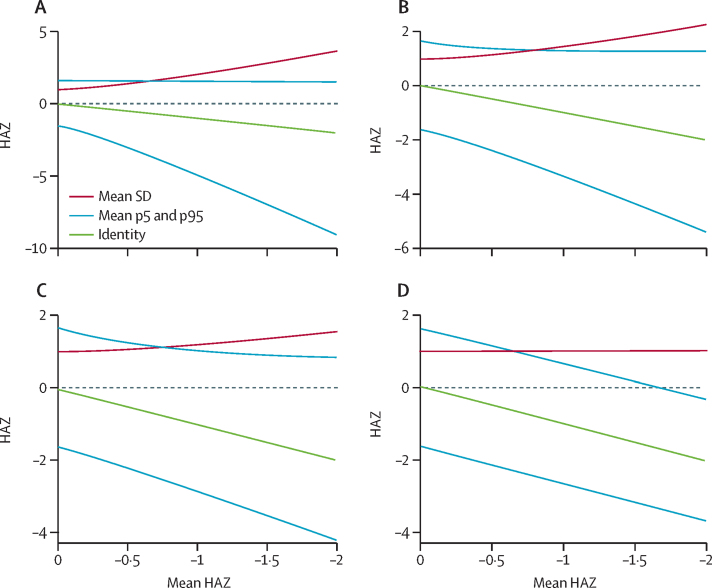
Simulations to demonstrate the theoretical effect of a set of growth-limiting exposures on standard deviation, fifth percentile, and 95th percentile of HAZ distribution for a simulated population of 10 000 children with an initial mean HAZ=0 and SD=1 (A) 25% of the population exposed; average standard deviation when mean HAZ=–2 was 3·63. (B) 50% of the population exposed; average standard deviation when mean HAZ=–2 was 2·25. (C) 75% of the population exposed; average standard deviation when mean HAZ=–2 was 1·55. (D) 100% of the population exposed; average standard deviation when mean HAZ=–2 was 1·02. Monte Carlo simulation (1000 repetitions) was used to simulate a faltering process in which the mean HAZ declined from 0 to −2 via cumulative 0·1 decrements in the fixed group of exposed children. Lines represent smoothed trends of the average standard deviation, 5th percentile, and 95th percentile. HAZ=height-for-age mean *Z* score.

Simulations showed that if faltering is limited to a minority of the population, there is a progressive widening and negative skewing of the HAZ distribution (figure 3A), with more pronounced widening and negative skewing of the HAZ distribution if the exposed subgroup preferentially includes children with low baseline HAZ (appendix pp 18–19). If faltering occurs in 50% of children, the distribution widens symmetrically (figure 3B). If a minority of children are protected against faltering, they would become progressively taller with age relative to their peers, leading to widening and positive skewing of the distribution (figure 3C). If all children are exposed to the same causes of faltering, mean HAZ declines without an increase in the SD or skewing of the upper or lower tail of the distribution (figure 3D). In further simulations, the introduction of a floor effect did not induce a narrowing of the HAZ distribution as mean HAZ declined (appendix pp 20–21). However, in simulated scenarios in which the risk of exposure was increased at higher baseline HAZ and there was a heterogeneous exposure distribution (ie, less than 100% of the population exposed), there was a transient narrowing of the distribution (appendix pp 22–23); or, if there was whole-population exposure (100% of the population exposed) but the magnitude of faltering was associated with higher baseline HAZ, the HAZ distribution narrowed as the mean declined (appendix pp 24–25).

## Discussion

Across 179 surveys in 64 countries, the postnatal decline in mean HAZ in low-income and middle-income countries was accompanied by declines in fifth and 95th percentile, and a symmetrical convergence of both tails of the HAZ distribution towards the mean. The downward shift of the distribution with age indicated that children across the entire HAZ spectrum experience postnatal linear growth faltering in low-income and middle-income countries. Because the decline in mean HAZ cannot be attributed to faltering of a high-risk subgroup of children within each population, postnatal linear growth faltering in low-income and middle-income countries should be considered a whole-population condition.

The observed shift of the HAZ distribution in the absence of negative skewness or widening of the distribution seemed to broadly reflect Geoffrey Rose's concept of whole-population shifts in distributions of chronic disease risk factor; however, the decrease in the SD was inconsistent with Rose's precept that whole-population shifts entail the preservation of a constant distribution width.^17^, ^18^ The relatively rapid descent of 95th percentile (compared with mean HAZ and fifth percentile) suggested that the tallest children in each population regress more strongly towards their own population's declining mean HAZ than towards the WHO median (HAZ=0). We therefore considered the possibility that the decline in population mean HAZ is disproportionately driven by faltering among relatively taller children. This hypothesis implies that taller children in each survey were at disproportionately higher risk of exposure to growth-limiting factors or that taller children were more susceptible to the adverse effects of growth-limiting risk factors even if the whole population was exposed. However, we could not identify a biological rationale for this hypothesis and it seems to be in direct conflict with consistent previous observations that, in low-income and middle-income countries, taller children tend to have relatively better socioeconomic circumstances.^4^, ^19^ We believe that a more plausible explanation for the nearly symmetrical pattern of narrowing of the HAZ distribution is regression to the mean caused by differential measurement error in HAZ by age, operating independently of the causes of faltering. Assaf and colleagues^20^ previously suggested that greater HAZ measurement error at younger ages explains the relatively wide distribution in early infancy and the decline in SD with age. We considered at least three possible sources of such differential measurement error: variation in gestational age at birth that is unaccounted for in the application of the WHO growth standards will increase HAZ variance in early infancy, and the effect diminishes with age; errors in maternal recall of birth dates would be more influential at younger ages; and measurement of height or length could yield relatively larger proportional errors in infants compared with toddlers.^21^ Notably, in longitudinal studies of infant cohorts in low-income and middle-income countries,^22^, ^23^ in which anthropometric measurement and age-ascertainment errors might be less problematic than in population-based surveys, SD seems to remain relatively constant as mean HAZ declines with age, which is in agreement with Rose's criteria. Therefore, although we cannot exclude the potential contribution of subgroup effects (eg, greater susceptibility of taller children to faltering) to the overall decline in mean HAZ and the change in the HAZ distribution with age, the present findings suggest that the dominant causes of postnatal linear growth faltering are community-wide factors to which nearly all children in the population are exposed. This inference strongly supports the emerging prioritisation of research and action on community-level determinants of child growth in low-income and middle-income countries (eg, nutrition-sensitive programmes).^1^, ^6^, ^7^, ^24^, ^25^

We acknowledge the practical difficulties in distinguishing individual-level versus community-level determinants of child growth.^8^, ^26^ Biological factors could be associated with HAZ along a continuous distribution of exposure,^27^ and therefore can be modelled in ways that show their associations with both child-level and population-average HAZ. For example, shorter mothers tend to give birth to shorter infants, and, across populations, low average maternal height could be associated with low average child HAZ. However, these associations have different interpretations because the causal pathways that underlie HAZ ranking of children within a population (eg, genetic factors that explain why a short mother will tend to have a short infant^9^, ^10^) differ from those that cause populations to deviate from the WHO distribution (eg, genetic variation does not cause substantial between-population differences in height distributions^2^, ^28^). Risk factors for stunting or low HAZ are often operationally defined to distinguish relatively worse versus better household characteristics or conditions (eg, unimproved versus improved sanitation in each household) based on variations within a community.^4^, ^19^, ^27^ However, causes of faltering might be child or household characteristics or behaviours that are ubiquitous or nearly homogeneous within a population and thus cannot be discerned as stunting risk factors due to a lack of between-child or between-household variation. Key community-level factors operate in the collective domain (eg, inadequate public sanitation infrastructure^29^) and would thus be unmeasurable at the individual child or household level. Generating evidence regarding ubiquitous or intrinsically community-level exposures is challenging,^6^, ^24^ because these factors require multipopulation datasets and multilevel analyses;^8^, ^26^ it is particularly difficult to gather high-quality reliable data on public policies, service provision, or environmental conditions^19^, ^29^, ^30^ compared with the social and economic descriptors that are frequently ascertained in household surveys.^7^, ^27^, ^31^ For example, the link between macroeconomic growth and stunting remains fiercely debated,^7^, ^32^, ^33^ partly because national income is not a precise surrogate of the policies and public services that influence the HAZ distribution.^7^ Ultimately, the notion that the whole population falters despite between-child variations in health and wealth remains perplexing. Assmann and Hermanussen^34^ argued that interpersonal proximity itself might drive the historical clustering of height within populations, but they were unable to pinpoint the biological mechanisms to explain this effect.^34^

Although empirical evidence that faltering in low-income and middle-income countries occurs across the entire HAZ spectrum has long been available (eg, from birth cohort studies^22^), chronic childhood linear growth faltering has only occasionally been explicitly acknowledged to be a whole-population condition rather than a discrete condition affecting a subgroup of children.^7^, ^35^, ^36^, ^37^ We are unaware of previous studies focused specifically on the changes in the HAZ distributional parameters associated with postnatal linear growth faltering. Mei and colleagues^38^ previously reported a lack of a relationship between SD and mean HAZ across DHS surveys; however, they did not quantitatively assess the change in SD with the age-related decline in mean HAZ, and they interpreted their findings only as a justification for using SD as an indicator of anthropometric survey quality. In previous multi-DHS studies, both Mei and colleagues^38^ and Assaf and colleagues^20^ noted that HAZ SDs are smaller in older versus younger children, a finding also observed in a survey in Tibet.^37^ A key strength of our study was the analysis of within-survey age-related variations in mean HAZ to generate inferences that were not confounded by causes of survey-level variations in mean HAZ and SD. Also, we used simulations to show that whole-population downward shifts in the HAZ distribution are more likely to be attributable to population-wide exposures rather than the differential experience of a high-risk subgroup.

We acknowledge several limitations of our cross-sectional analysis of survey data. Age-related differences in the HAZ distributions could be confounded by period effects; however, given that the different age groups in each survey were born over a period of only 3 years, changes in social and environmental conditions affecting the HAZ distributions were probably minor. Survey sizes varied greatly, so there was low precision of the values (particularly fifth percentile and 95th percentile) generated in some small age bands; however, our inferences were unchanged when analyses were limited to large surveys. DHS surveys variably suffer from problems with anthropometric data quality (ie, missing data, implausible values, or digit preference);^20^, ^38^, ^39^ the observed decline in SD with age was probably an artifact of age-differential measurement errors. It is also possible that DHS surveys disproportionately excluded the highest-income households in many countries; therefore, we use the term population to indicate the great majority of children in low-income and middle-income countries but acknowledge that a small proportion of children (<5%) in these settings might be non-faltering outliers. We only analysed HAZ rather than raw height values, which would have been difficult to interpret because between-child height variance normally increases with age when using raw values.^2^ Although our results strongly suggest that faltering is largely a whole-population condition, we could not prove that the narrowing of the HAZ distribution with age was attributable to age-differential measurement errors rather than within-population heterogeneity (ie, greater risk of faltering among children with higher HAZ at baseline). Moreover, even if faltering is a whole-population phenomenon, it does not imply that between-child variation in size or growth velocity is unimportant or immutable. We observed SDs that were consistently greater than in the reference population (ie, SD=1), which has been previously attributed to a combination of measurement error^20^, ^38^ and heterogeneity in the survey sample.^20^ However, HAZ over-dispersion (ie, SD>1) might also reflect excessive HAZ inequality in low-income and middle-income countries, indicating potentially remediable between-child variations in size that are independent of age-related faltering. Furthermore, within communities, children with relatively low HAZ are at increased risks of morbidity, mortality, and neurodevelopmental impairment,^1^ indicating the role of policies and programs that target children with low HAZ to reduce the risk of adverse health outcomes.

In summary, our analysis of population-based surveys from low-income and middle-income countries strongly suggests that postnatal linear growth faltering is a whole-population condition. Virtually all children in such settings experience growth restriction compared with international norms. The present findings indicate that early-life growth faltering in low-income and middle-income countries is a much greater public health problem than is currently suggested by focusing on the subset of children who meet standard anthropometric criteria for stunting. In the consideration of strategies to prevent linear growth faltering, research and action on community-wide determinants of growth should be a dominant approach to improving the health and nutritional status of children worldwide.
