# Supplementary material for: Early childhood linear growth faltering in low-income and middle-income countries as a whole-population condition: analysis of 179 Demographic and Health Surveys from 64 countries (1993–2015)
Source: Lancet Glob Health. 2017 Nov 10;5(12):e1249–57. doi: 10.1016/S2214-109X(17)30418-7 (PMC5695758; doi:10.1016/S2214-109X(17)30418-7)
Supplement: Supplementary appendix [file mmc1.pdf]

THE LANCET
Global Health

Supplementary appendix

This appendix formed part of the original submission and has been peer reviewed. We post it as supplied by the authors.

Supplement to: Roth DE, Krishna A, Leung M, Shi J, Bassani DG, Barros AJD. Early childhood linear growth faltering in low-income and middle-income countries as a whole-population condition: analysis of 179 Demographic and Health Surveys from 64 countries (1993–2015). *Lancet Glob Health* 2017; **5:** e1249–57.

Supplementary Material

<u>Supplementary Methods</u>

1) Anthropometry and Standardization of Lengths/Heights

Supine length (<24 months of age) and standing height (24 months or older) were measured to the nearest 0.1 cm by trained field personnel using portable wooden boards according to standard anthropometric techniques[1]. DHS protocols include multiple quality control methods, although the anthropometric data quality varies widely[1]. Sex- and age-standardized HAZ scores were generated using the World Health Organization (WHO) child growth standards[2]. The day, month and year of birth and of assessment were used to calculate age. If month and year of birth were available but not the day of birth, the day of birth was set to 15. If the date of birth or assessment was unavailable, then the age in months as imputed by DHS was converted to age in days (months x 30.4375).

2) Random-intercept models to estimate international summary means and 95% confidence intervals for each distributional parameter within each age band (Aim #1)

We used random-intercept linear models without covariates to generate international summary means and 95% confidence intervals for each parameter, accounting for clustering of surveys within countries. Separate models were run for each 3-month age band and for each parameter. In describing the model below, we refer to mean HAZ as the dependent variable, but used a similar approach for other distributional parameters:

$$mean\ HAZ_{jk} = \beta_0 + \mu_{0k} + e_{jk}$$

where survey year j is nested in country k. β_0 is the fixed effect representing the overall mean HAZ across all surveys (for the particular age band). μ_{0k} is the country-level random intercept and e_{jk} is the error term at the level of the survey.

3) Multilevel linear models to estimate associations between mean HAZ and other distributional parameters (Aim #2)

In describing the model below, we refer to SD as the dependent variable, but used a similar approach for other distributional parameters:

$$SD_{ijk} = \beta_0 + \beta_1(-meanHAZ_{ijk}) + \mu_{1jk}(-meanHAZ_{ijk}) + \mu_{0jk} + \mu_{0k} + e_{ijk}$$

where survey-age band *i* is nested in survey year *j* that is nested in country *k*. β_0 is a fixed effect representing the overall mean SD when mean HAZ=0. β_1 is a fixed effect representing the overall mean difference in SD for a one-unit decline in mean HAZ, and μ_{1jk} is the survey-specific random slope for the association between SD and mean HAZ (i.e., accounts for between-survey variation around β_1). μ_{0jk} and μ_{0k} are survey year- and country-level random intercepts, respectively (i.e., account for survey- and country-level variation around β_0), and e_{ijk} is the error term at the level of the survey-age unit.

Models were estimated using unstructured covariance matrices for the random effects at the survey level. We did not use models that additionally included a random slope at the country level because they did not converge for all analyses; however, when they did converge, coefficients and residual variances were virtually unchanged from our main models (data not shown). For each of the random effects, we generated best linear unbiased predictions (BLUPS) for each survey to plot survey-specific slopes of the relationship between SD/p5/p95 and mean HAZ.

References

1. Assaf S, Kothari MT, Pullum T. An assessment of the quality of DHS anthropometric data, 2005-2014. Rockville, Maryland, USA: ICF International, 2015.
2. World Health Organization. WHO child growth standards : length/height-for-age, weight-for-age, weight-for-length, weight-for-height and body mass index-for-age : methods and development. Geneva: World Health Organization; 2006.

Table S1. Demographic and Health Surveys included in the study

#	Country	Year
1.	Albania	2008
2.	Armenia	2000
3.	Armenia	2005
4.	Armenia	2010
5.	Azerbaijan	2006
6.	Bangladesh	1996
7.	Bangladesh	1999
8.	Bangladesh	2004
9.	Bangladesh	2007
10.	Bangladesh	2011
11.	Bangladesh	2014
12.	Benin	1996
13.	Benin	2001
14.	Benin	2006
15.	Benin	2011
16.	Bolivia	1994
17.	Bolivia	1998
18.	Bolivia	2003
19.	Bolivia	2008
20.	Brazil	1996
21.	Burkina Faso	1998
22.	Burkina Faso	2003
23.	Burkina Faso	2010
24.	Burundi	2010
25.	Central African Republic	1994
26.	Cambodia	2000
27.	Cambodia	2005
28.	Cambodia	2010
29.	Cambodia	2014
30.	Cameroon	1998
31.	Cameroon	2004
32.	Cameroon	2011
33.	Chad	1996
34.	Chad	2004
35.	Chad	2014-15
36.	Colombia	1995
37.	Colombia	2000
38.	Colombia	2005
39.	Colombia	2010

#	Country	Year
40.	Comoros	1996
41.	Comoros	2012
42.	Congo Brazzaville	2005
43.	Congo Brazzaville	2011
44.	Congo, Democratic Republic	2007
45.	Congo, Democratic Republic	2013
46.	Cote d'Ivoire	1994
47.	Cote d'Ivoire	1998
48.	Cote d'Ivoire	2011
49.	Dominican Republic	1996
50.	Dominican Republic	2002
51.	Dominican Republic	2007
52.	Dominican Republic	2013
53.	Egypt	1995
54.	Egypt	2000
55.	Egypt	2005
56.	Egypt	2008
57.	Egypt	2014
58.	Ethiopia	2000
59.	Ethiopia	2005
60.	Ethiopia	2011
61.	Gabon	2000
62.	Gabon	2012
63.	Gambia	2013
64.	Ghana	1993
65.	Ghana	1998
66.	Ghana	2003
67.	Ghana	2008
68.	Guatemala	1995
69.	Guatemala	1998
70.	Guinea	1999
71.	Guinea	2005
72.	Guinea	2012
73.	Guyana	2009
74.	Haiti	1994
75.	Haiti	2000
76.	Haiti	2005
77.	Haiti	2012
78.	Honduras	2005
79.	Honduras	2011
80.	India	1998
81.	India	2005

#	Country	Year
82.	Jordan	1997
83.	Jordan	2002
84.	Jordan	2007
85.	Jordan	2012
86.	Kazakhstan	1995
87.	Kazakhstan	1999
88.	Kenya	1993
89.	Kenya	1998
90.	Kenya	2003
91.	Kenya	2008
92.	Kenya	2014
93.	Kyrgyzstan	1997
94.	Kyrgyzstan	2012
95.	Lesotho	2004
96.	Lesotho	2009
97.	Lesotho	2014
98.	Liberia	2007
99.	Liberia	2013
100.	Madagascar	1997
101.	Madagascar	2003
102.	Madagascar	2008-09
103.	Malawi	2000
104.	Malawi	2004
105.	Malawi	2010
106.	Maldives	2009
107.	Mali	1995
108.	Mali	2001
109.	Mali	2006
110.	Mali	2012
111.	Moldova	2005
112.	Morocco	2003
113.	Mozambique	1997
114.	Mozambique	2003
115.	Mozambique	2011
116.	Namibia	2000
117.	Namibia	2006
118.	Namibia	2013
119.	Nepal	1996
120.	Nepal	2001
121.	Nepal	2006
122.	Nepal	2011
123.	Nicaragua	1997

#	Country	Year
124.	Nicaragua	2001
125.	Niger	1998
126.	Niger	2006
127.	Niger	2012
128.	Nigeria	1999
129.	Nigeria	2003
130.	Nigeria	2008
131.	Nigeria	2013
132.	Pakistan	2012
133.	Peru	1996
134.	Peru	2000
135.	Peru	2005
136.	Peru	2007
137.	Peru	2008
138.	Peru	2009
139.	Peru	2010
140.	Peru	2011
141.	Peru	2012
142.	Rwanda	2000
143.	Rwanda	2005
144.	Rwanda	2010
145.	Rwanda	2014-15
146.	Sao Tome and Principe	2008
147.	Senegal	2005
148.	Senegal	2010
149.	Senegal	2012
150.	Senegal	2014
151.	Sierra Leone	2008
152.	Sierra Leone	2013
153.	Swaziland	2006
154.	Tajikistan	2012
155.	Tanzania	1996
156.	Tanzania	1999
157.	Tanzania	2004
158.	Tanzania	2010
159.	Tanzania	2015-16
160.	Timor Leste	2009
161.	Togo	1998
162.	Togo	2013
163.	Turkey	1993
164.	Turkey	1998
165.	Turkey	2003

#	Country	Year
166.	Uganda	1995
167.	Uganda	2000
168.	Uganda	2006
169.	Uganda	2011
170.	Uzbekistan	1996
171.	Zambia	1996
172.	Zambia	2001
173.	Zambia	2007
174.	Zambia	2013-14
175.	Zimbabwe	1994
176.	Zimbabwe	1999
177.	Zimbabwe	2005
178.	Zimbabwe	2010
179.	Zimbabwe	2015

Table S2. Demographic and Health surveys from 1993 to 2015 excluded from the study due to lack of anthropometric data, surveys were interim, or data access was restricted.

#	Country	Year
1.	Bangladesh	1993
2.	Dominican Republic	1999
3.	Egypt	2003
4.	Eritrea	1995
5.	Eritrea	2002
6.	Guatemala	2008
7.	Indonesia	1994
8.	Indonesia	1997
9.	Indonesia	2002
10.	Indonesia	2007
11.	Indonesia	2012
12.	Jordan	2009
13.	Madagascar	2008
14.	Mauritania	2000-01
15.	Pakistan	2006
16.	Peru	2004
17.	Peru	2006
18.	Philippines	1993
19.	Philippines	1998
20.	Philippines	2003
21.	Philippines	2008
22.	Philippines	2013
23.	Senegal	1997
24.	South Africa	1998
25.	Ukraine	2007
26.	Vietnam	1997
27.	Vietnam	2002
28.	Yemen	1997
29.	Yemen	2013

Table S3. Means, standard deviations, 5th percentiles, and 95th percentiles of height-for-age z-score (HAZ) distributions in three-month age bands from 0 to 35 months of age, averaged across 179 demographic and health surveys from low- and middle-income countries (n=2,148 survey-age units)

Age band[1]	Number of children per survey, median (min, max)	Mean HAZ, Mean (95% CI)	Standard deviation, Mean (95% CI)	5th percentile, Mean (95% CI)	95th percentile, Mean (95% CI)	Change from <3-month age band		
						Mean HAZ	5th percentile	95th percentile
0 to <3m	276 (26, 2473)	-0.44 (-0.54, -0.34)	2.11 (2.00, 2.22)	-3.89 (-4.14, -3.64)	2.91 (2.67, 3.15)	0	0	0
3 to <6m	282 (24, 2683)	-0.51 (-0.61, -0.41)	2.00 (1.89, 2.11)	-3.75 (-4.01, -3.50)	2.64 (2.40, 2.88)	-0.07	0.14	-0.27
6 to <9m	280 (27, 2373)	-0.68 (-0.79, -0.56)	1.95 (1.86, 2.04)	-3.77 (-4.01, -3.54)	2.41 (2.21, 2.61)	-0.24	0.12	-0.50
9 to 12m	261 (29, 2162)	-0.91 (-1.02, -0.80)	1.91 (1.81, 2.01)	-3.94 (-4.18, -3.70)	2.26 (2.00, 2.52)	-0.47	-0.05	-0.65
12 to <15m	277 (29, 2527)	-1.18 (-1.30, -1.07)	1.89 (1.79, 1.98)	-4.08 (-4.28, -3.87)	1.86 (1.62, 2.10)	-0.74	-0.19	-1.05
15 to <18m	256 (32, 2502)	-1.41 (-1.54, -1.28)	1.89 (1.79, 1.99)	-4.29 (-4.52, -4.06)	1.79 (1.51, 2.08)	-0.97	-0.40	-1.12
18 to <21m	251 (20, 2372)	-1.63 (-1.77, -1.49)	1.80 (1.72, 1.89)	-4.42 (-4.62, -4.21)	1.39 (1.14, 1.63)	-1.19	-0.53	-1.52
21 to <24m	238 (38, 1939)	-1.71 (-1.85, -1.56)	1.78 (1.70, 1.85)	-4.50 (-4.71, -4.28)	1.15 (0.96, 1.35)	-1.27	-0.61	-1.76
24 to <27m	273 (32, 2370)	-1.62 (-1.75, -1.48)	1.76 (1.68, 1.84)	-4.35 (-4.55, -4.16)	1.37 (1.16, 1.59)	-1.18	-0.46	-1.54
27 to <30m	252 (36, 2464)	-1.74 (-1.88, -1.60)	1.68 (1.60, 1.75)	-4.46 (-4.66, -4.25)	1.03 (0.84, 1.22)	-1.30	-0.57	-1.88
30 to <33m	241 (28, 2331)	-1.77 (-1.92, -1.61)	1.66 (1.58, 1.74)	-4.47 (-4.69, -4.25)	0.93 (0.72, 1.15)	-1.33	-0.58	-1.98
33 to <36m	231 (28, 2143)	-1.79 (-1.93, -1.64)	1.65 (1.58, 1.72)	-4.47 (-4.68, -4.25)	0.84 (0.66, 1.02)	-1.35	-0.58	-2.07

[1] Age band assignment of each child was based on age in days, whereby 1 month=30.4375 days; therefore, the youngest age band is from 0 to 91.31 days.

Table S4. Estimates of the associations between distributional parameters and mean of the height-for-age z-score (HAZ) distributions in sensitivity analyses.

Sensitivity analysis	# of survey-age units	Estimated mean change in parameter (95% confidence interval) for a 1-unit decline in mean HAZ			
		Standard deviation	Median	Δ5th percentile	Δ95th percentile
Restricted to <24 months, using 3-month age bands	1,432	-0.15 (-0.18, -0.13)	-1.00 (-1.01, -0.99)	-0.27 (-0.33, -0.21)	-0.24 (-0.30, -0.18)
Excluding HAZ values <-6 or >6	2,148	-0.11 (-0.13, -0.10)	-1.02 (-1.02, -1.01)	-0.24 (-0.27, -0.20)	-0.16 (-0.20, -0.12)
Based on 1-month age bands	6,444	-0.19 (-0.21, -0.17)	-0.95 (-0.96, -0.94)	-0.20 (-0.24, -0.15)	-0.41 (-0.46, -0.36)
Based on 6-month age bands	1,074	-0.21 (-0.23, -0.18)	-0.99 (-1.00, -0.98)	-0.32 (-0.38, -0.26)	-0.28 (-0.34, -0.23)

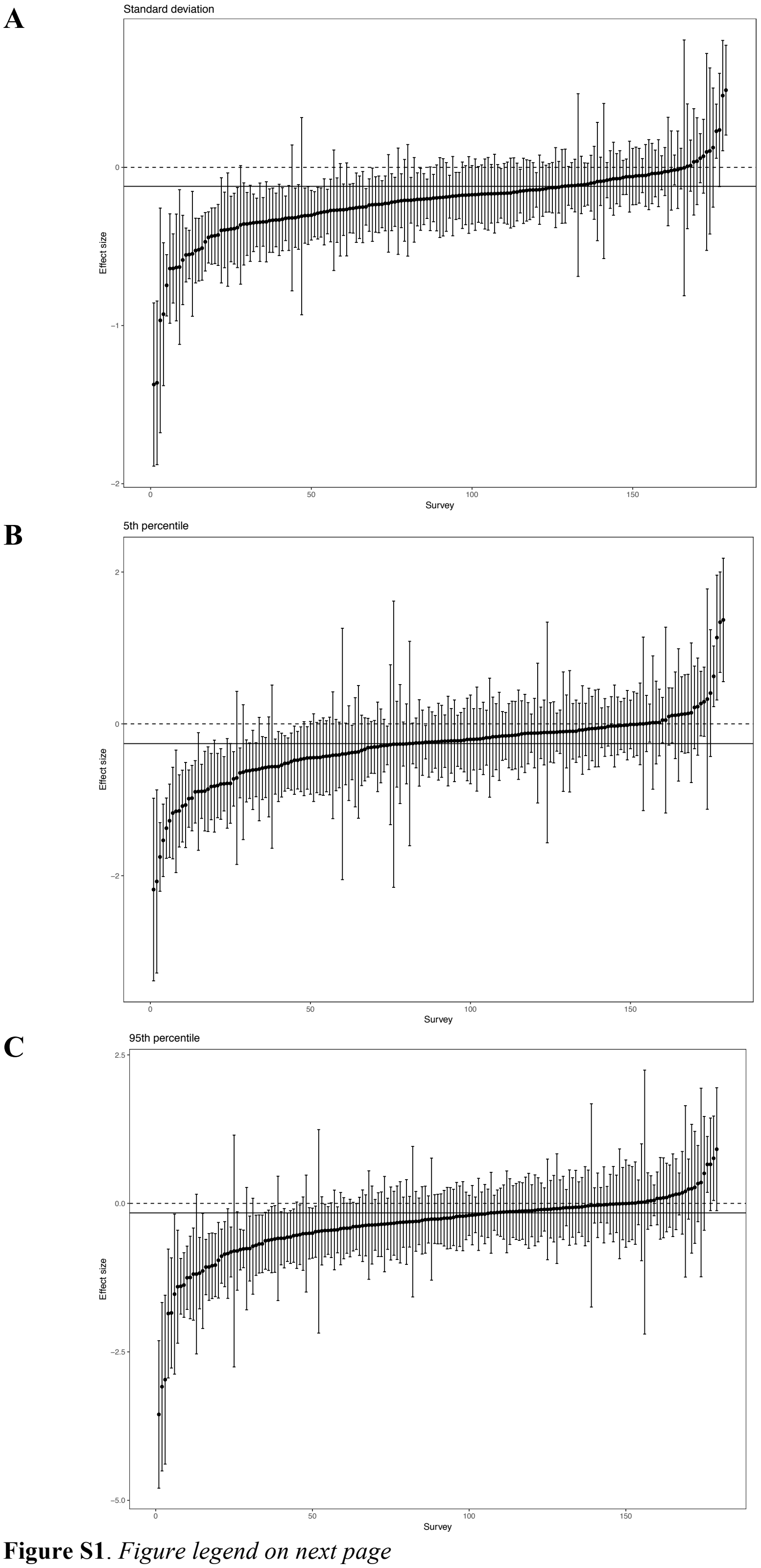

Figure S1. *Figure legend on next page*

Figure S1. Forest plots of 179 survey-specific estimates of the associations (and 95% confidence intervals) between the standard deviation (A), 5th percentile (B), and 95th percentile (C) of the height-for-age z-score (HAZ) distribution and mean HAZ. Estimates were based on fixed effects linear regression models that included interactions between survey and mean HAZ. Solid horizontal lines indicate the estimate of the grand mean slope from the corresponding multi-level model. Dashed horizontal lines indicate 0. In each panel, surveys are ordered along the x-axis by ascending magnitude of the survey-specific point estimate. For the association between standard deviation and mean HAZ (panel A), three surveys had point estimates and 95% confidence intervals above 0:

- Uzbekistan 1996: 0.23 (95% CI: 0.06, 0.40), n=1024;
- Sao Tome and Principe 2008: 0.45 (95% CI: 0.10, 0.80), n=1190;
- Guyana 2009: 0.49 (95% CI: 0.20, 0.77), n=1185.

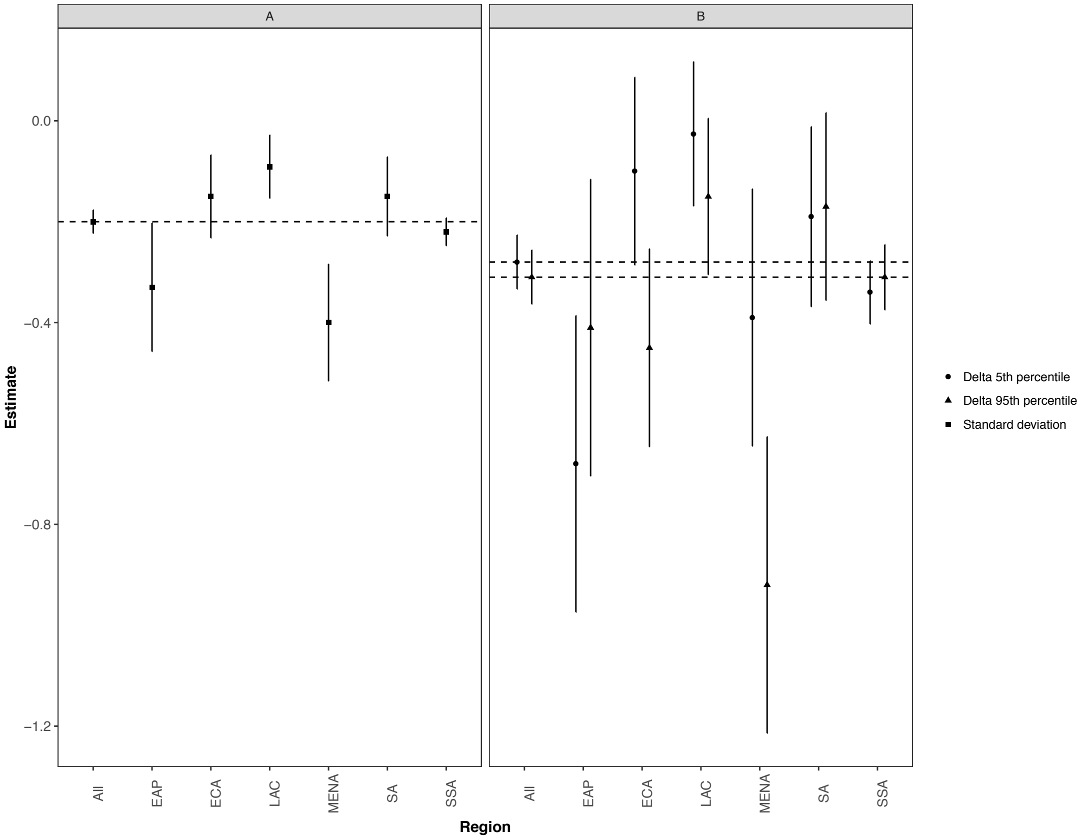

Figure S2. Estimates (and 95% confidence intervals) of the associations between the standard deviation (A), distance from mean to 5th percentile (B), and distance from mean to 95th percentile (B) of the height-for-age z-score (HAZ) distribution and mean HAZ in surveys stratified by World Bank world region: EAP= East Asia and Pacific; ECA = Europe and Central Asia; LAC= Latin America and Caribbean; MENA=Middle East and North Africa; SA=South Asia; SSA=sub-Saharan Africa. The dashed horizontal lines correspond to the overall ("all") point estimates for slopes of the standard deviation (panel A), distance from mean to the 5th percentile (B, lower line), and distance from mean to the 95th percentile (B, upper line).

(B)

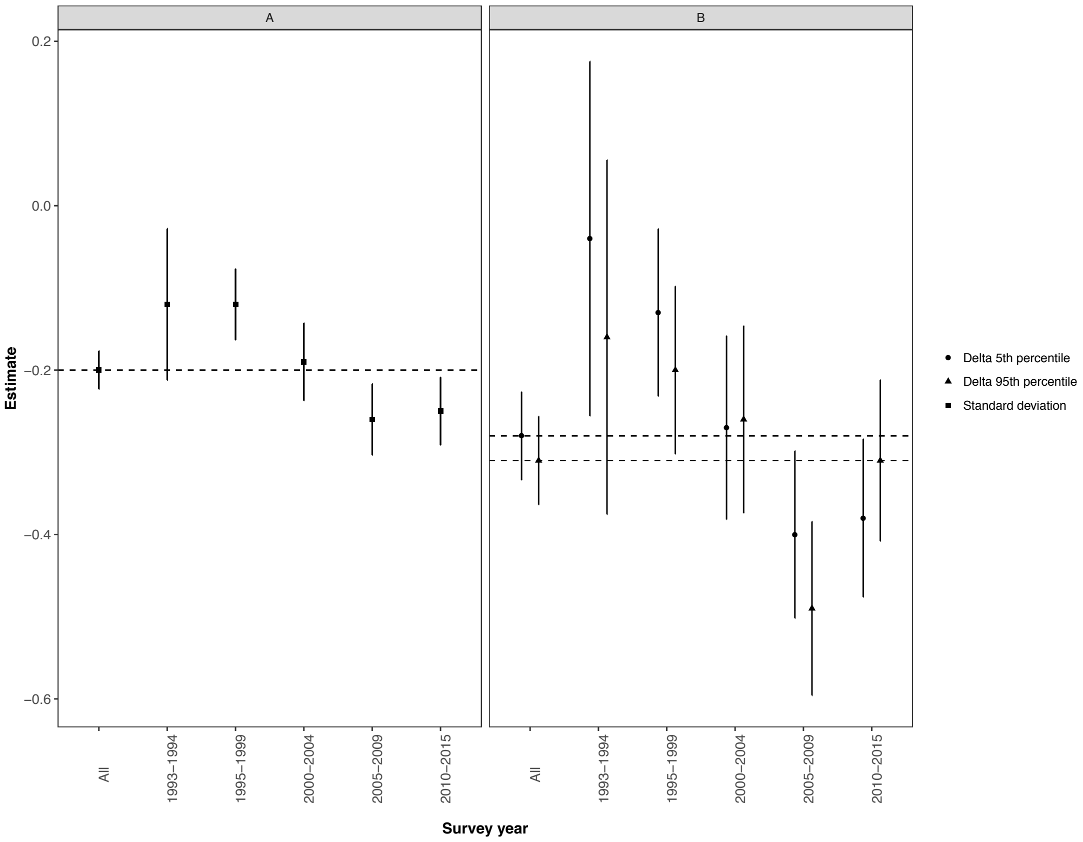

Figure S3. Estimates (and 95% confidence intervals) of the associations between the standard deviation (A), distance from mean to 5th percentile (B), and distance from mean to 95th percentile (B) of the height-for-age z-score (HAZ) distribution and mean HAZ in surveys stratified by survey year period. The dashed horizontal lines correspond to the overall ("all") point estimates for slopes of the standard deviation (panel A), distance from mean to the 5th percentile (B, lower line), and distance from mean to the 95th percentile (B, upper line).

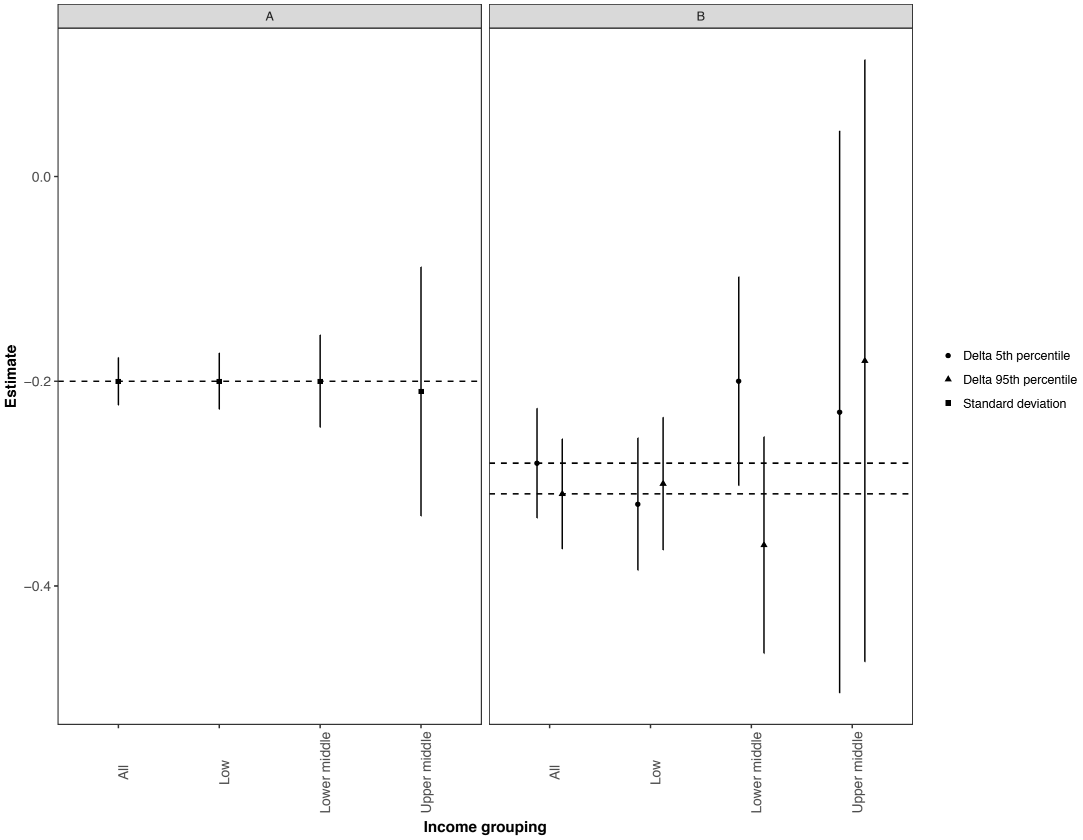

Figure S4. Estimates (and 95% confidence intervals) of the associations between the standard deviation (A), distance from mean to 5th percentile (B), and distance from mean to 95th percentile (B) of the height-for-age z-score (HAZ) distribution and mean HAZ in surveys stratified by World Bank income grouping. The dashed horizontal lines correspond to the overall ("all") point estimates for slopes of the standard deviation (panel A), distance from mean to the 5th percentile (B, lower line), and distance from mean to the 95th percentile (B, upper line).

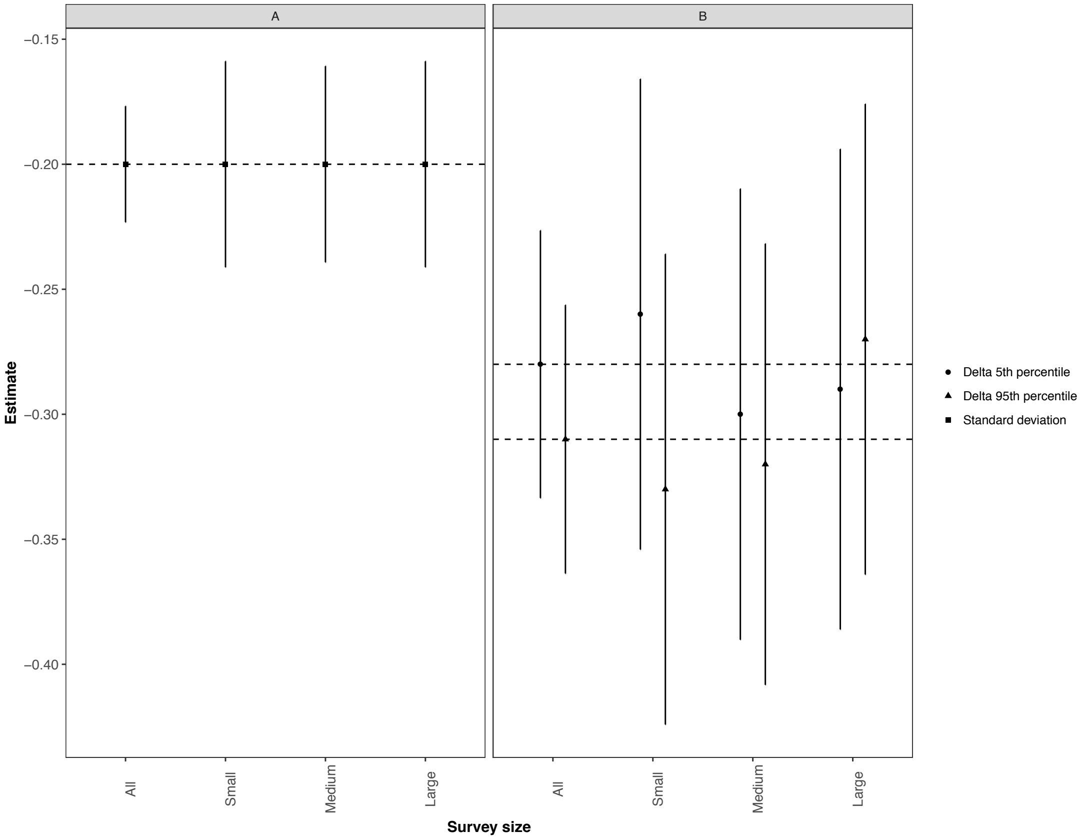

Figure S5. Estimates (and 95% confidence intervals) of the associations between the standard deviation (A), distance from mean to 5th percentile (B), and distance from mean to 95th percentile (B) of the height-for-age z-score (HAZ) distribution and mean HAZ in surveys stratified by survey size tertiles: Small: 351 to 2,471; Medium: 2,557 to 3,813; Large: 3,824 to 27,352. The dashed horizontal lines correspond to the overall ("all") point estimates for slopes of the standard deviation (panel A), distance from mean to the 5th percentile (B, lower line), and distance from mean to the 95th percentile (B, upper line).

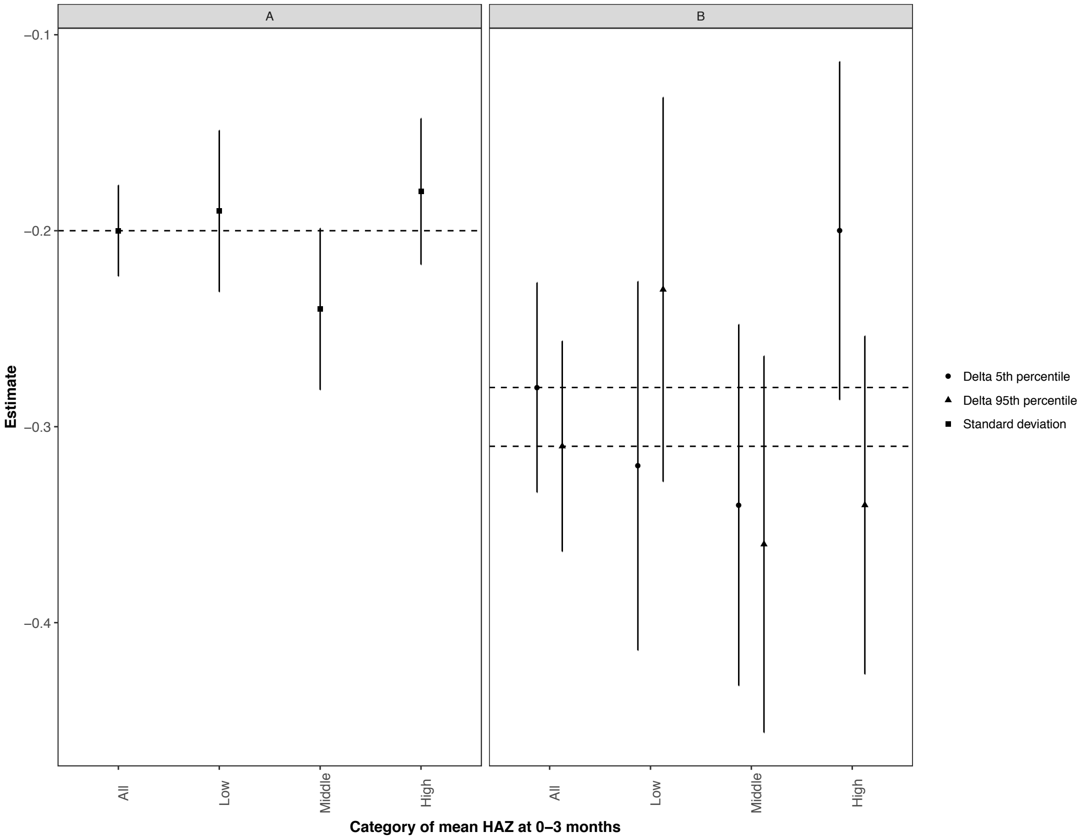

Figure S6. Estimated associations (and 95% confidence intervals) between the standard deviation (A), distance from mean to 5th percentile (B), and distance from mean to 95th percentile (B) of the height-for-age z-score (HAZ) distribution and mean HAZ in surveys stratified by tertiles of mean HAZ in the youngest age band (0 to <3 months):
Low: -2.07 to -0.58; Middle: -0.58 to -0.31; High: -0.31 to 0.79.
The dashed horizontal lines correspond to the overall ("all") point estimates for slopes of the standard deviation (panel A), distance from mean to the 5th percentile (B, lower line), and distance from mean to the 95th percentile (B, upper line).

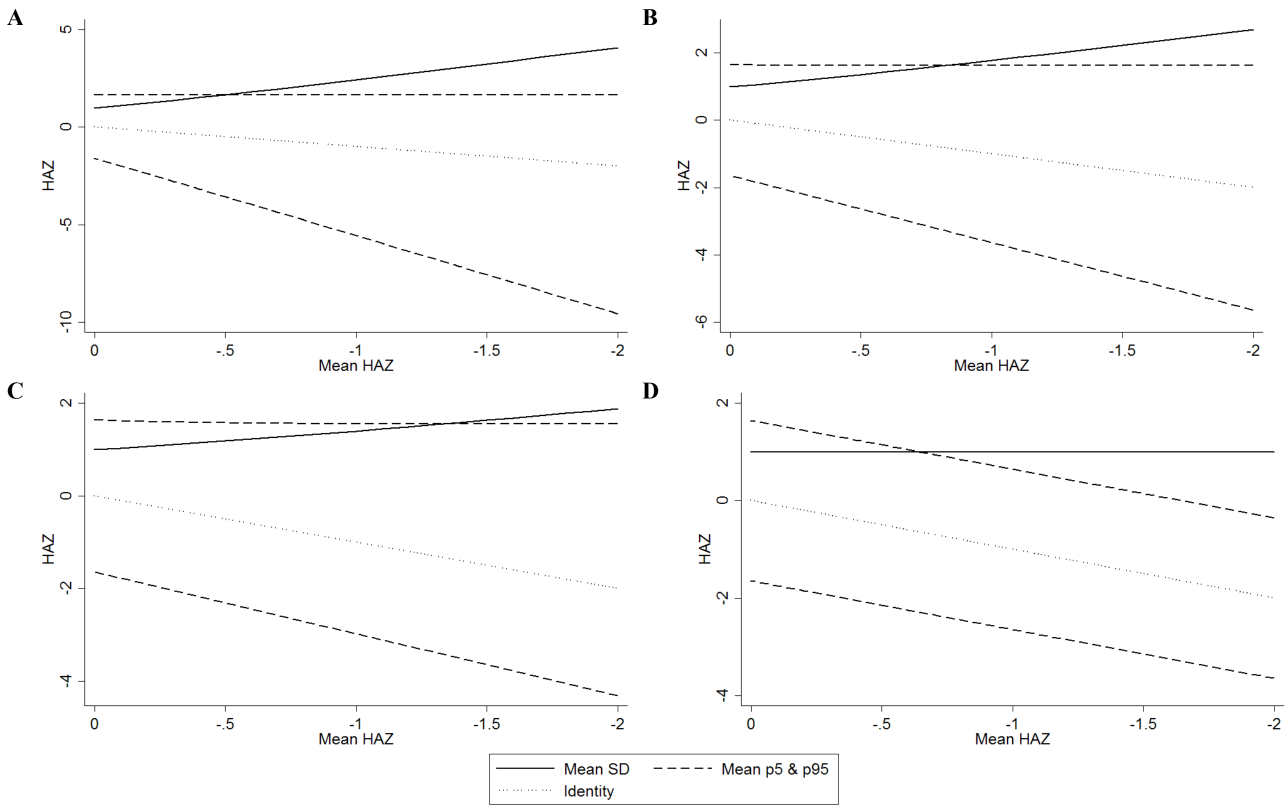

Figure S7. *Figure legend on next page*

Figure S7. Simulations with a higher risk of exposure at lower HAZ. The effect of a set of growth-limiting exposures on the standard deviation (SD), 5th percentile (p5) and 95th percentile (p95) of the height-for-age z-score (HAZ) distribution was simulated in a population of 10,000 children with an initial mean HAZ=0 and SD=1. Monte Carlo simulation (1000 repetitions) was used to simulate a faltering process in which the mean HAZ declined from 0 to -2 via cumulative 0.1 decrements in a fixed group of 'exposed' children. Lines represent smoothed trends of the average SD, p5 and p95. Scenarios A-D differ only with respect to the proportion of the population that was selected to be exposed to the set of growth-limiting factors (A=25%, B=50%, C=75%, D=100%).

Children with lower HAZ at the start of the simulation were preferentially selected into the exposure group (e.g., in Scenario A, we selected the 2,500 children with the lowest HAZ values).

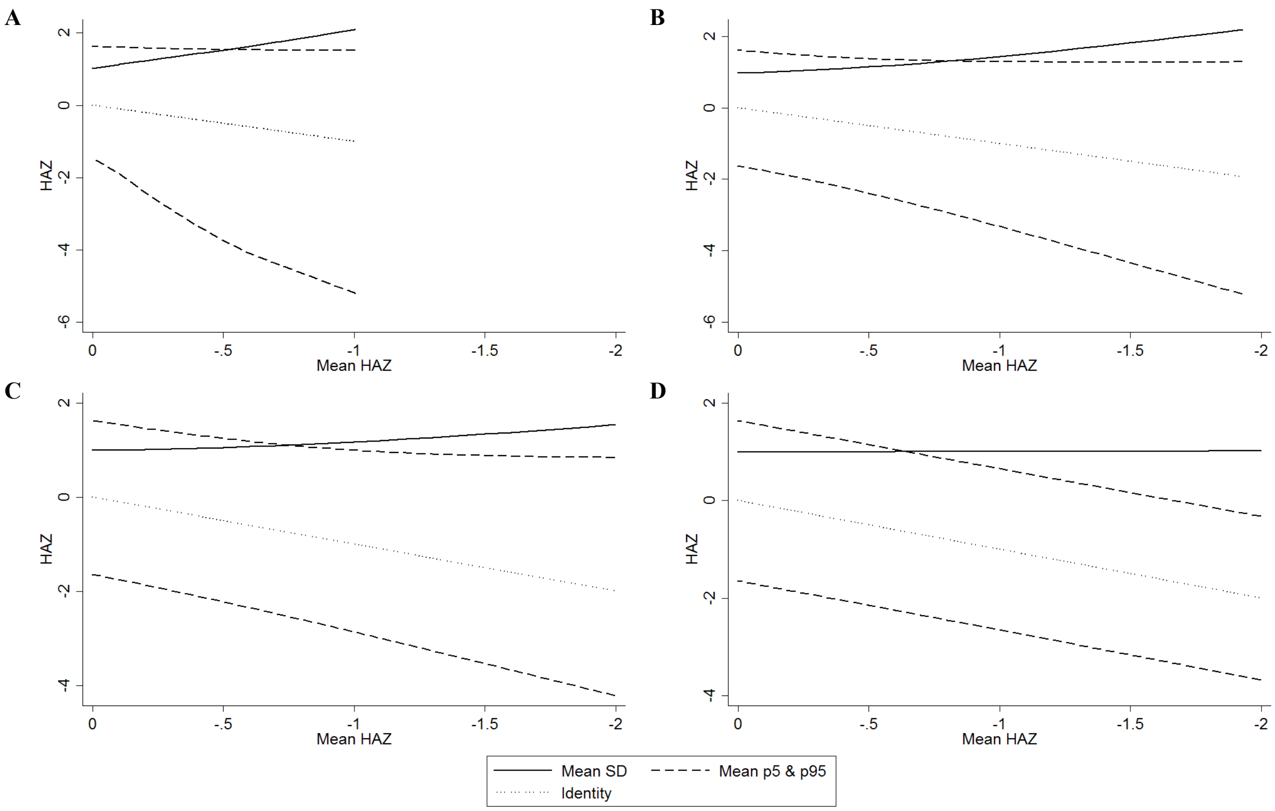

Figure S8. *Figure legend on next page*

Figure S8. Simulations with a floor effect. The effect of a set of growth-limiting exposures on the standard deviation (SD), 5th percentile (p5) and 95th percentile (p95) of the height-for-age z-score (HAZ) distribution was simulated in a population of 10,000 children with an initial mean HAZ=0 and SD=1. Monte Carlo simulation (1000 repetitions) was used to simulate a faltering process in which the mean HAZ declined from 0 to -2 via cumulative 0.1 decrements in a fixed group of 'exposed' children. Lines represent smoothed trends of the average SD, p5 and p95. Scenarios A-D differ only with respect to the proportion of the population that was randomly selected to be exposed to the set of growth-limiting factors (A=25%, B=50%, C=75%, D=100%).

A floor effect was imposed, whereby those children with HAZ<-6 were simulated to have died or been censored. In Panels A and B, the simulation ended prior to mean HAZ=-2 when all exposed children had HAZ <-6 (and therefore mean HAZ could no longer decline).

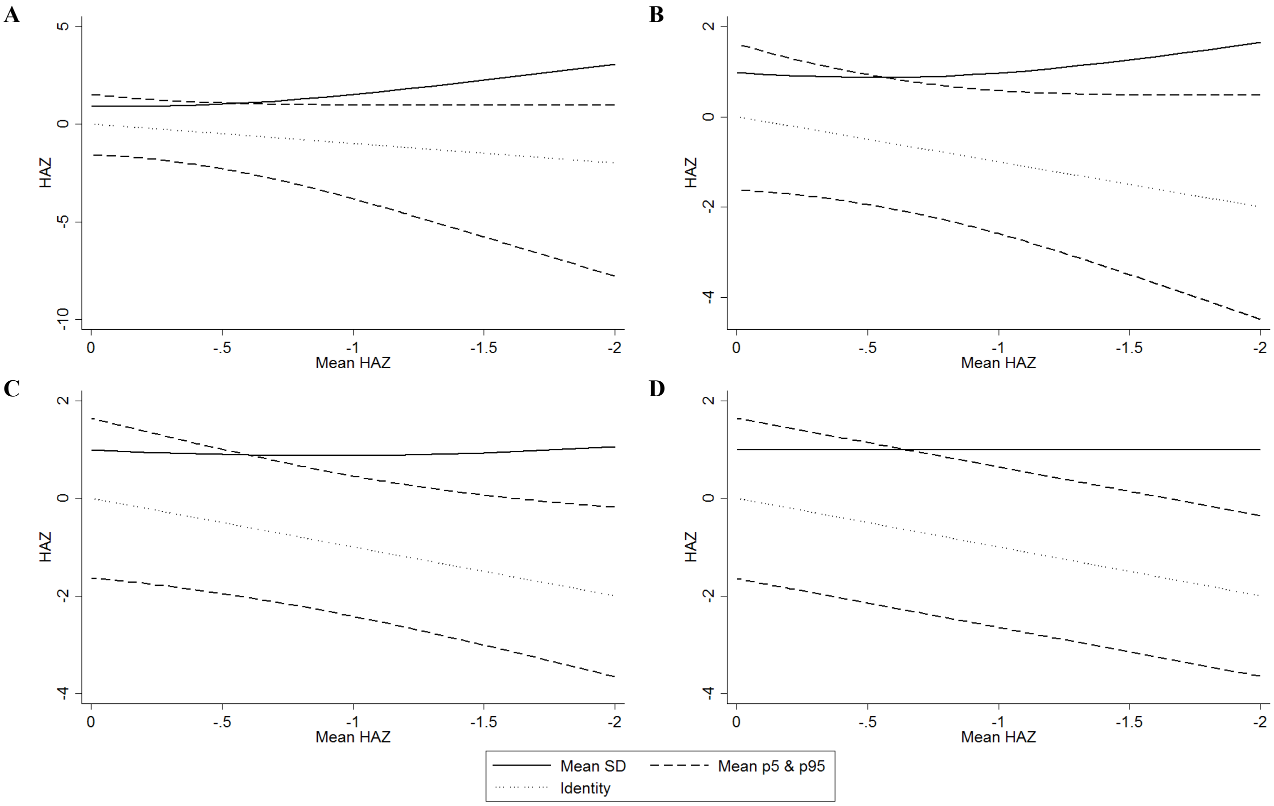

Figure S9. *Figure legend on next page*

Figure S9. Simulations with a higher risk of exposure at higher HAZ. The effect of a set of growth-limiting exposures on the standard deviation (SD), 5th percentile (p5) and 95th percentile (p95) of the height-for-age z-score (HAZ) distribution was simulated in a population of 10,000 children with an initial mean HAZ=0 and SD=1. Monte Carlo simulation (1000 repetitions) was used to simulate a faltering process in which the mean HAZ declined from 0 to -2 via cumulative 0.1 decrements in a fixed group of 'exposed' children. Lines represent smoothed trends of the average SD, p5 and p95. Scenarios A-D differ only with respect to the proportion of the population that was selected to be exposed to the set of growth-limiting factors (A=25%, B=50%, C=75%, D=100%).

Children with higher HAZ at the start of the simulation were preferentially selected into the exposure group (e.g., in Scenario A, we selected the 2,500 children with the highest HAZ values).

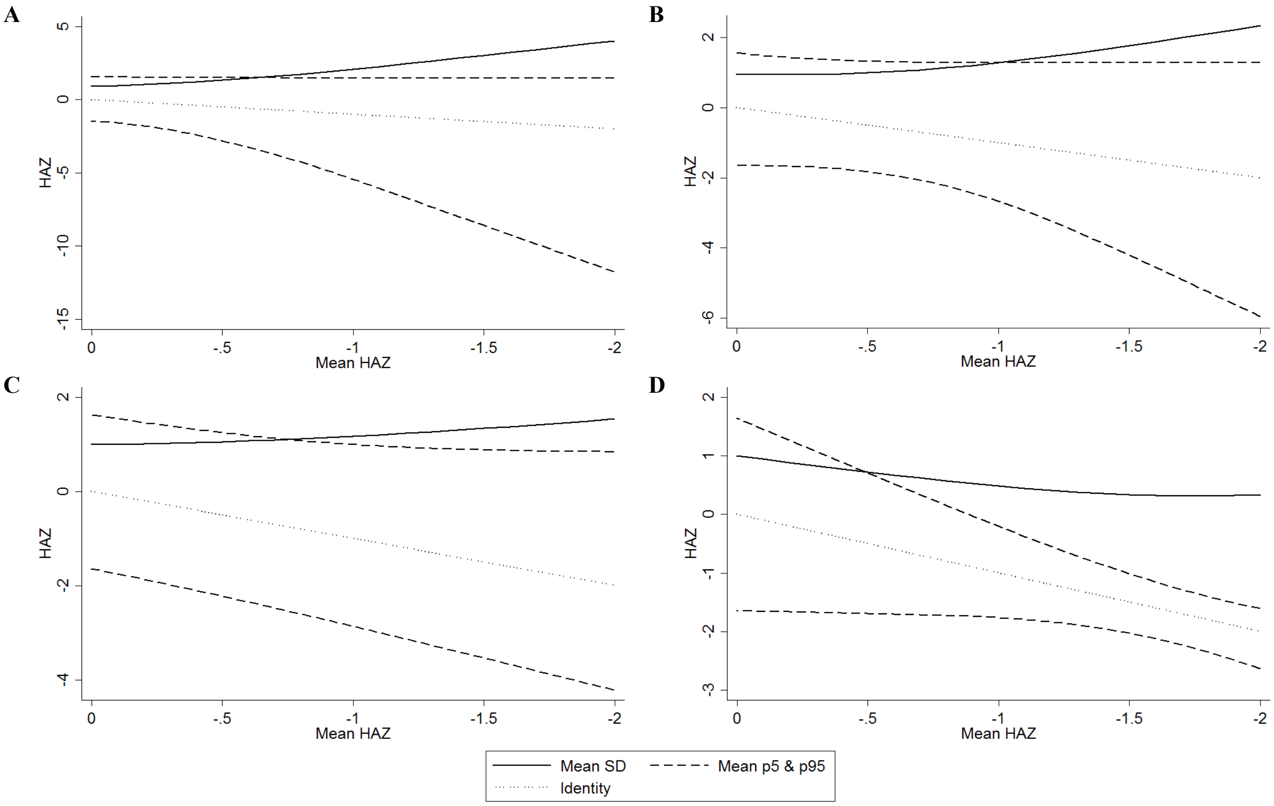

Figure S10. *Figure legend on next page*

Figure S10. Simulations with a higher magnitude of HAZ deficit for children with higher baseline HAZ. The effect of a set of growth-limiting exposures on the standard deviation (SD), 5th percentile (p5) and 95th percentile (p95) of the height-for-age z-score (HAZ) distribution was simulated in a population of 10,000 children with an initial mean HAZ=0 and SD=1. Monte Carlo simulation (1000 repetitions) was used to simulate a faltering process in which the mean HAZ declined from 0 to -2 via cumulative 0.1 decrements in a fixed group of 'exposed' children. Lines represent smoothed trends of the average SD, p5 and p95. Scenarios A-D differ only with respect to the proportion of the population that was randomly selected to be exposed to the set of growth-limiting factors (A=25%, B=50%, C=75%, D=100%).

The HAZ distribution at baseline was converted to an inverse normal probability distribution; in the exposed group, each child's probability value was used to calculate the proportional deficit incurred at each step of the simulation. Therefore, children with higher HAZ at the start of the simulation had relatively higher-magnitude decrements.

Lines represent smoothed trends of the average SD, p5 and p95.
